# Comparative Pathobiology of Fungal Pathogens of Plants and Animals

**DOI:** 10.1371/journal.ppat.1002324

**Published:** 2011-12-15

**Authors:** Martin B. Dickman, Paul de Figueiredo

**Affiliations:** 1 Borlaug Advanced Research Center, Texas A&M University, College Station, Texas, United States of America; 2 Department of Plant Pathology & Microbiology, Texas A&M University, College Station, Texas, United States of America; 3 Department of Veterinary Pathobiology, Texas A&M University, College Station, Texas, United States of America; 4 Department of Microbial and Molecular Pathogenesis, Texas A&M Health Science Center, College Station, Texas, United States of America; Duke University Medical Center, United States of America

Pathogenic fungi constitute a vast and diverse kingdom of eukaryotic organisms that interact with an equally vast and diverse collection of hosts. Despite this extraordinary diversity, unrelated fungi have strikingly similar needs and interests: nutrient acquisition, growth, niche establishment, and reproduction. To support these activities, fungi have evolved remarkably sophisticated mechanisms for interacting with host organisms, and modulating the speed, timing, and magnitude of these interactions. In the case of opportunistic human fungal pathogens, these mechanisms have often emerged as a consequence of the expansion of specific gene families and clusters that confer flexibility in nutrient acquisition, host recognition, and adhesion. These evolutionary adaptations do not generally support the destruction of the host, but rather, modulate homeostasis in the host to the advantage of the fungus (Figure 1). This scenario is analogous to what happens in plant pathogens as well. Biotrophs, which do not kill their hosts and require living cells for growth, co-opt homeostasis in the host to create an advantage for the fungus. Understanding the mechanisms by which fungi modulate biological activities in both plant and animal hosts remains an area of significant research interest and practical importance. Here, we describe four themes that emerge from a consideration of common mechanisms by which plant and animal fungi resist, subvert, or evade host defenses to ultimately thrive.

**Figure 1:**
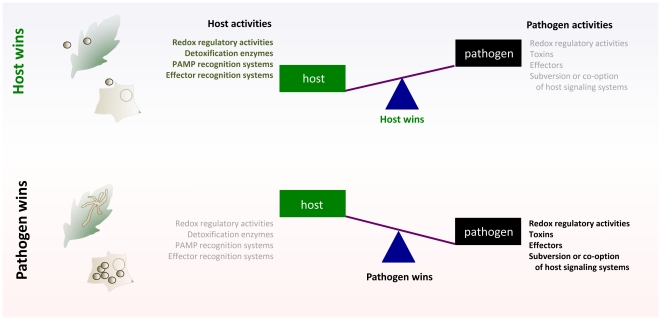
Tipping the balance for control in host–pathogen interactions; generalized schematic. To become established in plants or animals, fungal pathogens attempt to disrupt host cell homeostasis while avoiding and/or suppressing host recognition. The host has sophisticated surveillance systems that are poised to rapidly recognize non-self and counter disruptive attempts by pathogens. Signals activated by these surveillance systems can initiate a myriad of host defenses, including the release of reactive oxygen species and hydrolytic enzymes, which thwart the activities of fungal pathogens. The activation of host defense mechanisms also often culminates in the programmed death of host cells or tissue, which limits pathogen spread or dissemination. If attempts by the pathogen to co-opt, subvert, or avoid these host recognition and signaling mechanisms succeed, then the pathogen “wins” the battle for control of the interaction, and disease ensues. If the host wins this battle, then disease is averted.

## Toxins Are Not Always Toxic

Toxic metabolites produced by fungi and other microbes have been noted and characterized for more than a half century [1]. Treatment of host tissue with these compounds alone often recapitulates symptoms elicited by the pathogen. While toxins clearly can have harmful properties, our understanding of the precise manner by which toxins mediate pathogen virulence and/or compromise host defenses is in many cases incomplete. An emerging theme from various pathosystems suggests that the mode of action of several fungal toxins is based on the modulation of signaling pathways in the host as a means to achieve pathogenic success.

The broad host range necrotrophic fungal phytopathogen *Sclerotinia sclerotiorum* serves to illustrate. This fungus secretes the non-host selective toxin and key pathogenicity determinant oxalic acid (OA). This “simple” organic acid is toxic to host tissue, inducing cell death upon addition to various plants. However, *Sclerotinia* effectively uses OA for a range of processes including enzyme activation, guard cell regulation, and signaling for pathogenic (sclerotial) development. Importantly, these effects of OA on host tissue cannot be mimicked by treatment with other organic acids, including HCl, succinic acid, and citric acid [2]. Moreover, additional studies show that OA acts as a signaling molecule to induce a genetically regulated apoptotic-like programmed cell death (PCD) in host plant tissue [2]. Thus, the fungus tricks the host into generating nutrient-rich dead cells that are of sole and direct benefit to the fungus.

*Fusarium* spp. are rich sources of mycotoxins and other secondary metabolites. Fumonisin (FB1), for example, is a sphinganine analog and mycotoxin produced by *Fusarium verticillioides*, a maize endophyte associated with stalk rot disease. This toxin alters sphingolipid biosynthesis [3], modulates protein kinase C activity [4], [5], and also promotes disease in livestock and humans when ingested. Intriguingly, fumonisin induces apoptosis in human kidney cells [6], and in tomato and corn cells [7]. However, the toxin does not appear to be required for disease progression in plants. Rather, this toxin, and other fungal metabolites, may function to protect the fungus from predators and competitors in its environmental niche [8]. The activities of mycotoxins produced by some fungal pathogens of animals also possess unexpected activities. For example, *Aspergillus fumigatus*, the causative agent of aspergillosis in humans, produces the non-ribosomal immunotoxic dipeptide gliotoxin. This compound exerts pleiotropic effects in host tissue, including inhibiting macrophage phagocytosis, T cell proliferation, and mast cell activation [9].

## Oxygen: Can't Live with It, Can't Live without It

Everything must be in balance. This is both a philosophical tenet and a biological fact of life. The outcomes of host–microbe interactions are dependent, at least in part, on oxygen homeostasis. Membrane perturbation, a common early event in plant/animal–pathogen interactions, results in the induction and accumulation of reactive oxygen species (ROS), which include superoxide radicals (O_2_^−^), hydrogen peroxide (H_2_O_2_), and hydroxyl radicals (OH^.^). Thus, both host and pathogen must adapt quickly to changing environmental conditions or risk death. As a result, microbes, plants, and animals devote a significant portion of their cellular workload to tightly controlling their intracellular and perhaps extracellular redox environments [10] through the synthesis, regulation, and release of antioxidants (both enzymatic and non-enzymatic), which buffer and balance a potentially unstable environment [11].

This induction and accumulation of ROS, often called the oxidative or respiratory burst, is generally mediated by NADPH oxidases. In animals, the oxidative burst constitutes an important weapon used by host cells to eradicate pathogens following engulfment by macrophages. In humans, for example, defects in superoxide anion lead to the development of chronic granulomatous disease, an illness characterized by recurrent life-threatening bacterial and fungal infections [12]. In plants, the oxidative burst is an early, universal response to microbial attack that is correlated with several defense responses ranging from direct microbial toxicity to defense-associated enzyme activation, cell wall reinforcement, and the hypersensitive response (HR), a PCD that restricts pathogen spread. On the other hand, the detoxification or suppression of host-generated ROS is regarded as a defense mechanism by which pathogens evade the host immune response and survive inside hosts. In several fungal pathogens of plants and animals, the expression of redox homeostasis regulators (e.g., catalase, which detoxifies H_2_O_2_) has been implicated in counteracting the respiratory burst by protecting cells from death resulting from oxidative stress. It is also notable that fungi undergo oxidative bursts during development [13]. For instance, the oxidative burst is crucial for endophytes such as *Epichloe festucae* to establish symbiotic relationships with its plant host, rye grass [14], and also for sclerotial development in *S. sclerotiorum* [2] and for vegetative growth and development in *Neurospora crassa* [15].

Oxidative homeostasis is thus a focal point in host–pathogen interactions. Organisms have adapted to the presence and generation of ROS and have evolved processes to manipulate the oxidative environment by perturbing the balance between production and scavenging. When this balance is tipped, disease often occurs.

## Dirty Deeds

While plants and animals share several strategies for combating attacks by fungal pathogens, plants do not possess antibody-mediated immunity, and thus rely on innate mechanisms for immune defense. These mechanisms exploit Toll-like receptors (TLRs), pattern recognition receptors (PRRs), and receptor-like kinases (RLKs) that recognize pathogen-associated molecular patterns (PAMPs) to activate host defense programs. In the case of animals, these molecules also regulate phagocytosis [16]. The production of intercellular signaling molecules, such as pro-inflammatory mediators in the case of animal infection, and the plant hormones jasmonic acid, ethylene, and salicylic acid in the case of plant infection, also accompany pathogen recognition.

Not surprisingly, pathogenic fungi have co-evolved mechanisms to evade host defenses. These dirty deeds include the secretion of components that mask extracellular PAMPs and the synthesis of physical barriers and other molecules that suppress phagocytic recognition, uptake, and killing [17]. In addition, pathogenic fungi can direct the reprogramming of metabolic and signaling networks to evade or subvert host defenses [18]. Some pathogenic fungi, including *Candida albicans* [19] (human) and *Cladosporium fulvum* (tomato), also release metabolites that impair host innate immune function [20]. However, despite these subversive activities, these fungal pathogens cannot always successfully evade or circumvent host defenses.

## Death Means Life

Plants mount physical and chemical responses to attacks by fungal pathogens. They thicken their cell walls, produce anti-microbial compounds, and trigger HR, a process resulting in the delimitation of pathogen spread via activation of PCD at the site of infection. Thus, plants in essence altruistically sacrifice a few cells for the sake of the whole. However, certain plant pathogens can manipulate these cell death pathways to enhance plant colonization and promote disease. Besides *Sclerotinia*, the necrotrophic plant pathogen *Botrytis* spp. induces host cell death to secure nutrients [21]. Moreover, several toxin-producing fungi also impact PCD pathways. For example, *Cochliobolus victoriae* produces the host selective toxin victorin, which induces an apoptotic-like PCD and plant defense responses by targeting specific host proteins [22]. The complex polysaccharide galactoxylomannan, produced by the animal pathogen *Cryptococcus neoformans*, activates host apoptotic pathways through interactions with glycoreceptors on T cells, which, in turn, contribute to the immunosuppression that accompanies cryptococcosis [23], [24], [25]. *C. albicans* can also activate caspase-dependent apoptotic pathways during early stages of the infection process, which contributes to host cell death [26]. Thus, the manipulation of host cell death by fungal pathogens promotes disease progression and pathogen dissemination in fungal pathogens of plants and animals. Finally, it should be noted that some plant pathogens suppress PCD for pathogenic success. For example, the oomycete *Phytophthora infestans*, the causal agent of late blight in potato, expresses suppressor of necrosis 1 (SNE1) during biotrophic growth within host plants. This protein suppresses PCD signaling in the host and inhibits the PCD induction activities of its own proteins [27]. Bax inhibitor-1 (BI-1), identified in a screen for mammalian proteins that inhibit pro-apoptotic Bax, is one of few proteins conserved across kingdoms that impact PCD. BI-1 has been found in many plants, as well as yeast, and like its mammalian brethren, is a cytoprotective survival gene. This has not gone unnoticed by biotrophic fungi, where it has been shown that RNA interference of barley BI-1 resulted in plants that were less susceptible (more resistant) to powdery mildew than wild-type plants. Barley Bl-1 is targeted by the fungus and is required for complete susceptibility to barley powdery mildew fungus. Thus, barley BI-1 is viewed as a susceptibility factor for powdery mildew. We will likely see others in the future [28], [29]. In mammals, BI-1 expression is down-regulated as chronic liver damage progresses [30]. The high levels of mRNA observed in the early stages of liver disease may protect virus-infected cells against apoptosis, while progressive down regulation may facilitate hepatocellular carcinogenesis.

## Conclusions

The fight for control of a given host–pathogen interaction is not black and white, but rather highly context dependent. The speed, timing, and magnitude of the responses of the combatants, amid the lifestyle of the pathogen, all contribute to who wins the day in the battle during pathogen attack. Several strategies used by pathogens to get the upper hand are shared even though hosts are distinctly different. In the end, the outcome of interactions between fungal pathogens and their plant or animal hosts reduces to a simple question: Who's in control? If the pathogen or host wins the battle over the control of oxidative stress, PCD, and “defense” gene responses, that combatant will prevail.
